# Prostate artery embolization has long term efficacy for treatment of severe lower urinary tract symptoms from giant prostatic hyperplasia

**DOI:** 10.1186/s12894-020-00726-y

**Published:** 2020-10-08

**Authors:** Alexander S. Somwaru, Stephen Metting, Laura M. Flisnik, Michael G. Nellamattathil, Arjun Sharma, Venkat S. Katabathina

**Affiliations:** 1 Department of Diagnostic, Molecular, and Interventional Radiology, Icahn School of Medicine At Mount Sinai, 1000 10th Avenue, New York, NY 10019 USA; 2 Valley Radiology Medical Associates, San Jose, CA USA; 3 Department of Radiology, New York Presbyterian/Weill Cornell Medical Center, New York, NY USA; 4 Department of Radiology, MedStar Georgetown University Hospital, Washington, DC USA; 5 Department of Interventional Radiology, Brigham and Women’s Hospital, Boston, MA USA; 6 Department of Radiology, University of Texas Health Science Center San Antonio, San Antonio, TX USA

**Keywords:** Giant prostatic hyperplasia (GPH), Prostatic artery embolization (PAE), Lower urinary tract symptoms (LUTS)

## Abstract

**Background:**

Patients with severe lower urinary tract symptoms (LUTS) from giant prostatic hyperplasia (GPH): prostate volume greater than 200 mL that do not respond to medical therapy may not be eligible for surgical treatments due to morbidities, technical challenges, and patient preference. This retrospective investigation examined the long-term efficacy and safety of prostatic arterial embolization (PAE) as a treatment option for severe LUTS due to GPH in a large patient cohort.

**Methods:**

Of 529 patients who underwent PAE between January 2016 and January 2020, 72 patients had severe LUTS from GPH and were retrospectively evaluated. PAE was performed with two embolic agents in sequence: 100–250 μm particles followed by 2 mm and 3 mm coils. Clinical assessment was performed with international prostate symptoms score (IPSS), quality of life (QoL), peak flow rate (Qmax), post-void residual volume (PVR), and prostate specific antigen (PSA) measurements before and 12 months and 24 months after PAE. Prostate volume (PV) was measured by multiparametric magnetic resonance (MR) imaging before and 12 months and 24 months after PAE.

**Results:**

Patients with severe LUTS from GPH experienced significant clinical improvements in IPSS, QoL, Qmax, PVR, PSA, and PV at 12 months and 24 months after PAE. Mean IPSS decreased from 26.5 to 18.0 (*P* < 0.01) to 10.5 (*P* < 0.01). Mean QoL decreased from 6.0 to 4.0 (*P* < 0.01) to 2.0 (*P* < 0.01). Mean Qmax increased from 8.0 to 14 mL/s (*P* < 0.01) to 18 mL/s (*P* < 0.01). Mean PVR decreased from 198.0 to 152.0 mL (*P* < 0.01) to 90 mL (*P* < 0.01). Mean PV decreased from 303.0 mL to 258.0 mL (*P* < 0.01) to 209.0 mL (*P* < 0.01). Mean PSA decreased from 11.2 ng/mL to 9.5 ng/mL (*P* < 0.05) to 7.9 ng/mL (*P* < 0.05). No major complications occurred.

**Conclusions:**

PAE is a safe treatment with long term efficacy for severe LUTS from GPH. PAE may be a viable therapeutic option for patients with severe LUTS from GPH whom fail medical therapy and are not candidates for surgical treatments.

## Background

Patients with severe urinary tract symptoms (LUTS) from prostatic hyperplasia that is refractory to medical therapy are offered surgical treatment options that range from minimally invasive procedures to transurethral resection of the prostate (TURP) to simple prostatectomy [1–4]. The surgical treatment selection process is governed by prostate volume, presence of LUTS, comorbidities, risk–benefit profile, patient preference treatment availability and physician expertise [1–4]. Prostate volume is a significant factor in surgical treatment selection [1–4]. TURP and minimally invasive procedures, such as photoselective vaporization, laser enucleation, transurethral vapor and microwave ablation, transurethral incision, and prostate urethral lift, are best suited for patients with prostate volumes less than 80 mL [1–4]. Large prostatic hyperplasia is defined as prostate volume greater than 100 mL and giant prostatic hyperplasia (GPH) is defined as prostate volume greater than 200 mL [1–9]. Patients with large and giant prostatic hyperplasia with severe LUTS may require simple prostatectomy however may have a higher risk of morbidity from blood loss that requires blood transfusion (8–15%), urinary incontinence (10%), and urinary bladder neck stenosis and urethral strictures (5%) [1–9)].

Prostate artery embolization (PAE) has emerged as a safe and efficacious treatment of patients with severe LUTS from prostatic hyperplasia with prostate volumes up to 100 mL, or large prostatic hyperplasia [5–13]. Numerous retrospective and prospective studies of large cohorts of patients have shown that PAE safely provides long term reduction in symptoms and prostate volume by inducing ischemic necrosis, diminished hormonal response, and reduced prostate innervation with consequent reduced smooth muscle tone [10–13].

GPH is defined as prostate volume greater than 200 mL [1–9, 14–19]. GPH has been described in several individual patients as case reports in a review of international scientific literature [1–9, 14–19]. Individual cases of patients with severe LUTS from GPH whom underwent treatment with surgery or minimally invasive ablative urological treatment have also been reported [20–26]. However the safety and long-term efficacy of PAE for treatment of large cohorts of patients with severe LUTS from GPH has not been examined. One study retrospectively reviewed eight patients and one case report reviewed one patient whom underwent PAE for severe LUTS from GPH [27, 28]. While these two studies reported short term efficacy: eight months and three months, respectively, for significant symptom relief, long term efficacy remains unknown [27, 28]. A prostate volume limit that may render PAE ineffective, due to unsuccessful symptom and prostate volume reduction, has also not been established [1–4, 27, 28]. This study evaluated the safety and long-term efficacy of PAE in the largest cohort of patients, to date, with severe LUTS due to GPH.

PAE has a steep learning curve because it requires both refined microvascular skills and detailed knowledge of highly variable anatomic anatomy to identify, catheterize, and embolize target prostatic arteries to attain successful outcomes and avoid adverse complications [5–8]. The variant origins and anatomies of the prostate artery have been, in turn, reported in a variable fashion across anatomic and angiographic literature. The most commonly reported origins are the internal pudendal artery, the superior vesical artery (with a common trunk), inferior vesical artery, the gluteal-pudendal arterial trunk, and the obturator artery [5–11]. Prostate artery anatomy origins, number, accessory branches, and extra-prostatic anastomoses of the prostate artery may vary from left to right pelvic side. The prostatic artery bifurcates into the central and capsular divisions. The superior and anterolateral pedicle, which is the primary arterial target of PAE, provides the dominant supply to the central gland and the inferior and posterolateral pedicle provides supply to the peripheral gland and prostate apex [5–12]. In hyperplastic prostate glands, the prostate artery is tortuous with intraglandular twists and spiral arterial branches, which are used as landmarks for angiographic identification and confirmation of catheter position during PAE [5–13]. Extra-arterial anastomoses commonly communicate with the internal pudendal artery, contralateral prostate artery, and superior and inferior vesical arteries [5–10, 12].

Clinical volume and experience allow the operator to develop a level of expertise in PAE, which is a technically challenging although safe and efficacious procedure, however complications may occur. PAE is performed under direct fluoroscopic guidance with cone-beam CT. Cone-beam CT is particularly useful to assist in the identification of the prostatic arteries, reduce ionizing radiation dose and procedural time, and minimize complications of nontarget embolization. Non-target embolization of pelvic organs, like the urinary bladder, urethra, rectum, and penis, is a source of complications such a hematuria, rectal bleeding, erectile dysfunction, penile skin changes and pain that usually resolve without intervention or surgery [1–5]. Acute urinary retention and dysuria may be caused increased pressure and compression of the intraprostatic urethra from swelling, edema, ischemia, and necrosis of the prostate gland that occur after embolization that may require temporary post-procedural catheterization of the urinary bladder until spontaneous urination resumes [1–5]. Urinary tract infections, also thought to be caused by increased pressure and compression of the intraprostatic urethra from swelling, edema, ischemia, and necrosis of the prostate gland, require outpatient antibiotic therapy [1–5]. Other self-limited complications include hematospermia, urethral pain, and soft tissue hematomas at the arterial access site [1–5].

## Methods

Institutional review board (IRB) approval was received for this retrospective study. Written informed consent was obtained from the patients and any accompanying images. Patients gave consent for their personal or clinical details along with any identifying images to be published in this study. A review of the electronic medical records (EMR) from a single medical center was conducted on all patients (529) whom underwent PAE for severe LUTS between January 2016 and January 2020. Severe LUTS was classified as International Prostate Symptom Score (IPSS) greater than 18 points, urinary quality of life (QoL) greater than 3 points, and peak flow rate (Qmax) less than 12 mL/s. Patients were evaluated in this investigation if they underwent PAE for severe LUTS from GPH. GPH is defined as prostate volume greater than 200 mL. Patients that did not have GPH (prostate volumes less than 200 mL) were not evaluated in this investigation. Of the 529 patients that underwent PAE for severe LUTS, 72 patients had GPH and were evaluated in this investigation. At our institution, multiparametric MR imaging is performed on all patients before PAE for prostate volume (PV) measurements and to ensure the absence of any lesions suspicious for clinically significant cancers according to version 2.1 of Prostate Imaging-Reporting and Data System (PI-RADS) [29]. No patients underwent PAE if MR imaging revealed lesions suspicious for clinically significant cancer. MR imaging was also used to measure prostate volumes at 12 months and 24 months after PAE. Patients underwent PAE if they did not respond to 5-alpha-reductase inhibitor and/or alpha-1-adrenergic receptor antagonist medical therapy for at least 6 months or longer and were not eligible for surgery or refused surgery. Patients were not eligible for surgical treatment due to comorbidities and/or contraindications to surgery and/or general anesthesia 49 (68%) patients with cardiovascular disease on anticoagulation/antiplatelet therapy and 23 (32%) patients with chronic lung disease. IPSS, QoL, Qmax, PV, postvoid residual volume (PVR), and PSA were measured and collected before PAE and 12 months and 24 months after PAE. Because MR imaging is performed on all patients before PAE to ensure the absence of any lesions suspicious for clinically significant cancers in our institution, patients with PSA levels above 4 ng/mL were examined in this investigation. Before PAE, 5-alpha-reductase inhibitor therapy was stopped and alpha-1-adrenergic receptor antagonist therapy was tapered and stopped after one month after PAE. No patients underwent additional interventions after PAE.

### Clinical metrics

Clinical assessment was performed in all patients before PAE and 12 months and 24 months after PAE with history and physical examination, IPSS and QoL questionnaires, uroflowmetry Qmax, PVR, and PSA levels measurements. These clinical metrics are routinely recorded for patient care, to optimize patient outcomes, and for quality improvement and performance.

### Prostate MR imaging and volume measurement

The protocol at our institution is to perform multiparametric MR imaging for all patients before PAE for prostate gland volume measurements and to ensure the absence of clinically significant cancers before PAE. Prostate volumes were calculated using MR imaging obtained before PAE and 12 months and 24 months after PAE. At our institution, multiparametric prostate MR imaging is performed using 3.0 T magnet systems (Siemens Healthcare, Erlangen, Germany). Exams are performed with phased array torso coils using the following protocol (Table 1): axial, sagittal, and coronal T2-weighted turbo spin echo images; axial b50, b500, and b800 s/mm^2^ diffusion-weighted images; synthetic extrapolated b1200, b1500, b2000, and b2500 diffusion-weighted images; apparent diffusion coefficient (ADC) map; axial T1 pre-contrast fat saturated volumetric interpolated breath-hold examination (VIBE) images; coronal T1-precontrast fat-saturated MR angiographic VIBE images of the pelvis; serial dynamic axial T1 pre-contrast fat saturated VIBE images obtained after intravenous gadolinium contrast injection (Gadavist 0.1 mmol/kg; Bayer Healthcare Pharmaceuticals, Wayne, NJ, USA); axial fat-saturated T1-weighted delayed postcontrast VIBE images.

**Table 1:** MRI acquisition protocol

Sequence	Plane	Slice/gap (mm)	TR/TE (ms)	FOV (mm)	Matrix	Flip angle
T2W TSE	Sagittal	5/1	4000/100	200 × 200	384 × 224	150
T2W TSE	Axial	3/0	4000/100	200 × 200	384 × 224	150
T2W TSE	Coronal	3/0	4000/100	200 × 200	384 × 224	150
DWI (b0, 50, 500, 800, 1200, 1500, 2000, 2500 s/mm^2^)/ADC	Axial	3/1	3300/60	260 × 260	128 × 96	
T1W FS VIBE MRA	Coronal	5/1	3.85/1.42	260 × 260	256 × 180	70
T1W FS VIBE pre	Axial	5/1	3.85/1.42	260 × 260	256 × 180	100
T1W FS VIBE post dynamic × 3	Axial	5/1	3.85/1.42	260 × 260	256 × 180	2.5/10/20
T1W FS VIBE post whole pelvis	Axial	5/1	3.85/1.42	260 × 260	256 × 180	70

*ADC* apparent diffusion coefficient, *DWI* diffusion weighted imaging, *FOV* field of view, *FS* fat saturated, *MRA* magnetic resonance angiography, *TE* echo time, *TR* repetition time, *TSE* turbo spin echo, *VIBE* volumetric interpolated breath-hold examination

Two diagnostic radiologists with 12 and 6 years of experience in interpreting multiparametric prostate MR imaging, respectively, independently reviewed the MR imaging exams before and after PAE. The radiologists used DynaCAD software (InVivo, Philips Healthcare, Amsterdam, Netherlands) on two separate workstations to perform semiautomated prostatic volumetric measurements of the prostate using the MR T2-weighted images. Prostatic volumes were manually confirmed by calculation of the ellipsoid volume formula (L × W × H × π/6). Discordant measurements were resolved by consensus agreement. The diagnostic radiologists also reviewed MR imaging before and after PAE for any prostate gland lesions suspicious for clinically significant cancer according to version 2.1 of Prostate Imaging-Reporting and Data System (PI-RADS) [29].

### Prostate artery embolization

All PAE procedures were performed by a single operator with thirteen years of angiographic and embolization experience and four years of experience performing PAE. All patients received one dose of ciprofloxacin 400 mg administered intravenously for infection prophylaxis. All procedures were performed under moderate (conscious) sedation with local anesthesia in a therapeutic angiography unit. A unilateral left trans-radial arterial approach was utilized in all patients. Real time ultrasound was used to visualize patency and access of the left radial artery, which entered with a micropuncture set, 21-gauge needle, and a 5-French (-F) sheath. Patients underwent digital subtraction angiography (DSA) and PAE with a digital flat-panel detector fluoroscopy system (Innova 4100 IQ; General Electric Healthcare, Chicago, IL, USA) with nonionic intravenous contrast (Omnipaque 350 mg/mL; General Electric Healthcare, Chicago, IL, USA). Internal iliac arterial angiography in the ipsilateral oblique projection was performed to identify the right and left prostatic arteries, accessory prostatic arteries, and variant anatomy. Pelvic and prostatic arteries were catheterized using a combination of wires and catheters: 5-F Cobra 2 catheter (Cook Medical, Bloomington, IN, USA), 4-F Berenstein catheter (Merit Medical Systems, Incorporated, South Jordan, UT, USA), 2.4-F Progreat microcatheter (Terumo Interventional Systems, Tokyo, Japan) and 0.014-inch Transend microguide wire (Stryker Neurovascular, Fremont, CA, USA). The prostatic arteries were identified with DSA. Advanced imaging was performed with localized intraoperative cone-beam computed tomography (CT) with intravenous contrast (100 mL Isovue 370, Bracco Diagnostics, Milan, Italy) prior to embolization. Cone-beam CT images were transmitted, reconstructed in three dimensions, and reviewed to confirm prostatic arterial vascular anatomy, prostate gland vascular supply, and ensure the absence of vascular supply to adjacent anatomical structures, such as the urinary bladder, penis, and rectum. Bilateral PAE was then performed to stasis with a primary embolic agent: 100–250 μm Embospheres (Merit Medical Systems, Incorporated, South Jordan, UT, USA) and a secondary embolic agent: 2 mm and 3 mm CX coils (Boston Scientific, Marlborough, MA, USA). A band compression device was utilized to achieve radial arterial vascular access closure in all patients.

DSA, which was performed with a flat panel detector fluoroscopy system, and cone-beam CT both utilize photoelectric x-rays with ionizing radiation to visualize, identify, and catheterize the prostatic arteries during PAE. In accordance with the American College of Radiology, European Union of Radiology, and Society of Interventional Radiology, ionizing radiation was used in a fashion as low as reasonably achievable (ALARA) to minimize exposure of patients to radiation [30]. Low radiation doses to patients were maintained and further reduced with intraprocedural cone-beam CT in combination with three dimensional reconstruction that in turn abbreviated procedure times [30].

The Quality Improvement Guidelines for Percutaneous Transcatheter Embolization were used to classify complications after PAE; major complications are complications that require inpatient treatment and/or surgery and minor complications are complications that can be treated with conservative and/or outpatient treatment [31].

### Statistical analysis

The clinical metrics of IPSS, QoL, Qmax, PVR, PV, and PSA were expressed as quantitative values with means and standard deviations (SD). These quantitative values were analyzed with a Wilcoxon signed rank test using SAS software, version 9.4 (SAS Institute Incorporated, Cary, NC, USA). A probability value of *P* < 0.05 or lower was considered statistically significant. We had no missing information for the patients and data that were presented in this study.

## Results

72 patients with GPH were included with a mean baseline prostate volume of 303 mL (range 201–644 mL). Mean patient age was 71 years old (range 53–89 years); 72 males; 34 patients identified as European descent, 21 patients identified as African descent, 12 patients identified as Asian descent, and 5 patients identified with various descents.

**Fig. 1:**
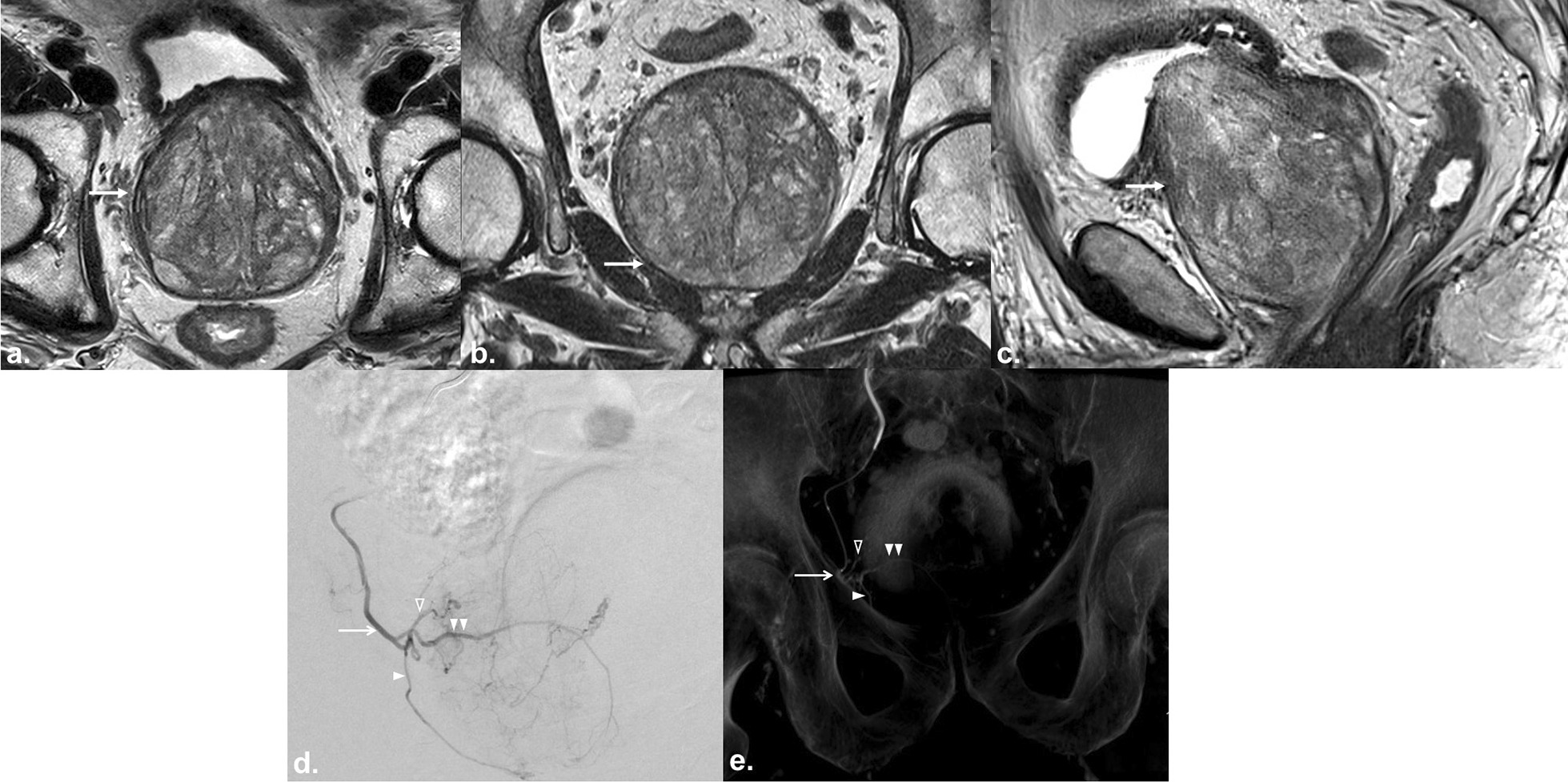
A 71 year-old patient with severe lower urinary tract symptoms (LUTS) from giant prostatic hyperplasia (GPH) underwent prostate artery embolization (PAE). **a** Axial T2-weighted turbo spin echo (TSE) image, **b** coronal T2-weighted TSE image, and **c** sagittal T2-weighted image from multiparametric magnetic resonance (MR) imaging show giant hyperplasia of the prostate gland (arrow) that measures 312 mL in volume. **d** Digital subtraction angiography (DSA) of selective catheterization of the right internal iliac artery anterior division shows a common origin of the right prostatic artery (straight arrow), which is hypertrophied, and the right superior vesical artery (open arrowhead). The anterior/lateral prostatic artery (single arrowhead) and the posterior/lateral prostatic artery (double arrowheads) are hypertrophied with a corkscrew pattern of the intraprostatic arterioles. **e** Cone-beam computed tomography (CT) with intravenous contrast in the coronal plane after selective catheterization of the internal iliac artery anterior division shows the anatomy of the right prostatic artery: a common origin of the right prostatic artery (straight arrow) and the right superior vesical artery (open arrowhead), hypertrophy of the anterior/lateral prostatic artery (single arrowhead) and the posterior/lateral prostatic artery (double arrowheads), and no vascular supply to the adjacent anatomical structures, to include the urinary bladder, penis, and rectum

Bilateral PAE was technically successful in all patients (Fig. 1: representative patient). Clinical metrics before PAE and 12 months and 24 months after PAE were collected (Table 2). Clinical metrics for each patient were retrieved from the EMR. Mean IPSS decreased from 26.5 ± 5.0 (SD) to 18.0 ± 4.5 (*P* < 0.0.01) 12 months after PAE and decreased from 18.0 ± 4.5 to 10.5 ± 4.0 (*P* < 0.01) 24 months after PAE. Mean QoL decreased from 6.0 ± 1.0 to 4.0 ± 1.0 (*P* < 0.01) 12 months after PAE and decreased from 4.0 ± 1.0 to 2.0 ± 1.0 (*P* < 0.01) 24 months after PAE. Mean Qmax increased from 8.0 mL/s ± 2.0 mL/s to 14 mL/s ± 5.0 mL/s (*P* < 0.01) at 12 months to 18 mL/s ± 4.0 mL/s (*P* < 0.01) at 24 months. Mean PVR decreased from 198.0 mL ± 20.0 mL to 152.0 mL ± 25.0 mL (*P* < 0.01) at 12 months and decreased from 152.0 mL ± 25.0 mL to 90 mL ± 15.0 mL (*P* < 0.01) at 24 months. Mean PV decreased from 303.0 mL ± 20.0 mL to 258.0 mL ± 15.0 mL (*P* < 0.01) at 12 months and decreased from 258.0 mL ± 15.0 mL to 209.0 mL ± 15.0 mL (*P* < 0.01) at 24 months. Mean PSA decreased from 11.2 ng/mL ± 2.5 ng/mL to 9.5 ng/mL ± 1.5 ng/mL (*P* < 0.05) at 12 months to 7.9 ng/mL ± 1.5 ng/mL (*P* < 0.05) at 24 months.

**Table 2:** Clinical metrics summary

Clinical metric	Before PAE Mean ± SD	12 months after PAE Mean ± SD	24 months after PAE Mean ± SD	*P* values
IPSS (points)	26.5 ± 5.0	18.0 ± 4.5	10.0 ± 4.0	< 0.01
QoL (points)	6.0 ± 1.0	4.0 ± 1.0	2.0 ± 1.0	< 0.01
Qmax (mL/sec)	8.0 ± 2.0	14.0 ± 5.0	18.0 ± 4.0	< 0.01
PVR (mL)	198 ± 20.0	152 ± 25.0	90 ± 15.0	< 0.01
PV (mL)	303 ± 20.0	258 ± 15.0	209 ± 15.0	< 0.01
PSA (ng/mL)	11.2 ± 2.5	9.5 ± 1.5	7.9 ± 1.5	< 0.05

*IPSS* International Prostate Symptom Score, *mL* milliliter, *PAE* prostate artery embolization, *PSA* prostate specific antigen, *PV* prostate volume, *PVR* postvoid residual volume, *Qmax* peak flow rate, *QoL* urinary quality of life, *SD* standard deviation

Minor complications occurred in 55 (76%) patients, resolved between two and seven days after PAE, and were conservatively managed. 20 (28%) patients experienced acute urinary retention that required temporary urinary bladder catheterization, 14 (19%) patients experienced urethral pain and dysuria, 11 (15%) patients experienced hematuria, eight (11%) patients experienced hemospermia, and two (3%) patients experienced small hematomas at the radial arterial access site. No major complications occurred.

No lesion suspicious for clinically significant cancer was detected on multiparametric MR imaging obtained in patients before or after PAE. All patients had PI-RADS v2.1 scores of PI-RADS 1: very low (clinically significant cancer is highly unlikely to be present) and PI-RADS 2: low (clinically significant cancer is unlikely to be present); no patient had scores of PI-RADS 3: intermediate (the presence of clinically significant cancer is equivocal), PI-RADS 4: high (clinically significant cancer is likely to be present), or PI-RADS 5: very high (clinically significant cancer is highly likely to be present).

## Discussion

Patients with LUTS due to prostatic hyperplasia that do not respond to medical therapy may be candidates for surgical and minimally invasive treatments. However patients with GPH have limited treatment options due to risks associated with these therapies. Simple prostatectomy, which is the gold standard, and minimally invasive urological procedures may have complications such as blood loss that requires transfusion, urinary incontinence, urinary bladder neck stenosis, and urethral strictures [5–8]. Moreover these surgical and minimally invasive treatments require general anesthesia, which has its own associated risks.

The current set of studies on the efficacy of PAE for treatment of LUTS from prostatic hyperplasia continues to grow. Our study can be added to the contemporary data. This investigation demonstrated the long term efficacy of PAE for treatment of severe LUTS from GPH: prostate volumes greater than 200 mL, in a large patient cohort at 12 months and 24 months after PAE. Mean value for IPSS, QoL, PVR, PV, and PSA significantly decreased and mean Qmax significantly increased at 12 months and at 24 months after PAE. The patients in our investigation continued to experience significant reductions in QoL, Qmax, PVR, and PSA after 12 months and at 24 months after PAE that may be potentially attributed to sustained long term hormonal downregulation and diminished neuromuscular feedback as well as greater initial prostate volumes (greater than 200 mL). These long term results are similar to prior investigations that illustrated the long term efficacy of PAE for treatment of LUTS due to prostate hyperplasia with prostate volumes 80–100 mL, in contrast to this investigation, which examined PAE treatment of LUTS from GPH with prostate volumes greater than 200 mL [5–13]. These studies demonstrated significant decreased severity of symptoms, improved quality of life, increased peak flow rates, and reduction in post void residual and prostate volumes and PSA levels [5–13]. Prior investigators have shown the efficacy of PAE in patients with LUTS from large prostatic hyperplasia: prostate volume greater than 100 mL however clinical outcomes specifically in patients with GPH, prostate volumes greater than 200 mL, have not been reported [5–13]. Bhatia et al. reported a single patient whom underwent PAE for treatment of severe LUTS from GPH had significant symptomatic relief at three months: IPSS decreased from 26.0 to 4.0, QoL decreased from 6.0 to 1.0, and PV decreased from 571 to 270 mL; PVR was not measured before PAE due to inability to void and was 50 mL after PAE.[27]. Mathevosian et al. showed similar success and short term efficacy in eight patients whom underwent PAE for severe LUTS from GPH and had sustained symptomatic relief at eight months: IPSS decreased from 20.5 to 3.8, QoL decreased from 4.4 to 1.4, and PV decreased from 318 to 212 mL [28]. However the limitations of these studies are small patient cohorts, one and eight, and short follow-up times, 3 months and 8 months [27, 28]. Larger cohorts of patients and long term efficacy have not been examined. Moreover an upper limit prostate volume for the efficacy of PAE has not been established. This upper limit volume number can be used to match patients to the appropriate therapy.

We confirmed that PAE is not only an effective treatment but also a safe therapy for severe LUTS in patients with GPH (prostate volumes greater than 200 mL) because no major complications occurred in 72 patients. No patient experienced a major complication, which required inpatient treatment and/or surgery. 76% of patients experienced at least one minor complication, which was either conservatively managed, treated in an outpatient or ambulatory fashion, or was self-limited and resolved. 20 (28%) patients experienced acute urinary retention that required temporary urinary bladder catheterization that was likely due to anticipated increased pressure and compression of the intraprostatic urethra from swelling, edema, and ischemic necrosis of the prostate gland after PAE. The urethral pain and dysuria in 14 (19%) patients, the hematuria in 11 (15%) patients, and the hematospermia in eight (11%) patients may have been caused by non-target embolization of the urethra and/or penis or passage of blood from ischemic necrosis of the prostate gland.

Our study has objective strengths with limitations. While we examined the largest number of patients whom underwent treatment of LUTS from GPH with PAE to date, results may be improved with larger cohorts. The length of follow-up time was longer than any other prior study: 24 months, which establishes sustainability, nevertheless longer-term follow-up studies may verify sustained patient outcomes. Our patients were referred from urologists and therefore subject to intrinsic referral bias. This bias was abated by blinding the interpreting radiologists to the indication for the multiparametric MR imaging exams. There were additional limitations to this investigation. This study was a single-institution, retrospective experience at a tertiary care academic medical center with urology, PAE, and MR availability and experience. Therefore our results may not be transferrable to all patient populations due to accessibility and contraindications. While no major complications occurred in 72 patients, this investigation was retrospective and therefore prospective safety may not be transitively established. PAE is a procedure that may not be widely available to all patients. Certain patients with LUTS from GPH may not be candidates for PAE. If a patient has a contraindication to receiving procedural anesthetic moderate sedation, then a patient cannot undergo PAE. Certain patients may have contraindications to undergo MR imaging, such as medical devices, hardware, or claustrophobia, for prostate volume measurement. In these patients, CT may be used for follow-up imaging for prostate volume measurement. Future research may be directed to prospective, multivariate comparisons of the efficacy of minimally invasive urological procedures and PAE in large patient cohorts for long follow-up times.

## Conclusions

PAE is an efficacious and safe treatment that provides significant and sustained relief of severe LUTS in patients with GPH. PAE may play an important role in the treatment patients with severe LUTS from GPH in whom medical therapy has failed, are not candidates for surgical and minimally invasive treatments, or refuse surgical treatment. We hope that these results, along with future investigations directed to prospective, multivariate, comparative and randomized studies, may provide patients with the best treatment to optimize symptom relief, minimize morbidity, and achieve safe and successful outcomes.

## Data Availability

The data sets used and analyzed during the current study are available from the corresponding author on reasonable request.

## Abbreviations

GPH: Giant prostatic hyperplasia
BPH: Benign prostatic hyperplasia
PAE: Prostatic artery embolization
LUTS: Lower urinary tract symptoms
IPSS: International Prostate Symptom Score
mL: Milliliter
PV: Prostate volume
PVR: Postvoid residual volume
QoL: QoL urinary quality of life
Qmax: Peak flow rate
PSA: Prostate specific antigen
MRI: Magnetic resonance imaging
ADC: Apparent diffusion coefficient
DWI: Diffusion weighted imaging
FOV: Field of view
FS: Fat saturated
MRA: Magnetic resonance angiography
TE: Echo time
TR: Repetition time
TSE: Turbo spin echo
VIBE: Volumetric interpolated breath-hold examination
F: French
DSA: Digital subtraction angiography
IRB: Institutional review board
EMR: Electronic medical records
HIPAA: Health Information Portability and Accountability Act
